# Genetic parameters for social effects on survival in cannibalistic layers: Combining survival analysis and a linear animal model

**DOI:** 10.1186/1297-9686-42-27

**Published:** 2010-07-07

**Authors:** Esther D Ellen, Vincent Ducrocq, Bart J Ducro, Roel F Veerkamp, Piter Bijma

**Affiliations:** 1 Animal Breeding and Genomics Centre, Wageningen University, Marijkeweg 40, 6709PG Wageningen, The Netherlands; 2 UMR 1313 GABI, INRA, 78352 Jouy-en-Josas, France; 3 Animal Breeding and Genomics Centre, Wageningen UR Livestock Research, 8200AB Lelystad, The Netherlands

## Abstract

**Background:**

Mortality due to cannibalism in laying hens is a difficult trait to improve genetically, because censoring is high (animals still alive at the end of the testing period) and it may depend on both the individual itself and the behaviour of its group members, so-called associative effects (social interactions). To analyse survival data, survival analysis can be used. However, it is not possible to include associative effects in the current software for survival analysis. A solution could be to combine survival analysis and a linear animal model including associative effects. This paper presents a two-step approach (2STEP), combining survival analysis and a linear animal model including associative effects (LAM).

**Methods:**

Data of three purebred White Leghorn layer lines from Institut de Sélection Animale B.V., a Hendrix Genetics company, were used in this study. For the statistical analysis, survival data on 16,780 hens kept in four-bird cages with intact beaks were used. Genetic parameters for direct and associative effects on survival time were estimated using 2STEP. Cross validation was used to compare 2STEP with LAM. LAM was applied directly to estimate genetic parameters for social effects on observed survival days.

**Results:**

Using 2STEP, total heritable variance, including both direct and associative genetic effects, expressed as the proportion of phenotypic variance, ranged from 32% to 64%. These results were substantially larger than when using LAM. However, cross validation showed that 2STEP gave approximately the same survival curves and rank correlations as LAM. Furthermore, cross validation showed that selection based on both direct and associative genetic effects, using either 2STEP or LAM, gave the best prediction of survival time.

**Conclusion:**

It can be concluded that 2STEP can be used to estimate genetic parameters for direct and associative effects on survival time in laying hens. Using 2STEP increased the heritable variance in survival time. Cross validation showed that social genetic effects contribute to a large difference in survival days between two extreme groups. Genetic selection targeting both direct and associative effects is expected to reduce mortality due to cannibalism in laying hens.

## Background

Mortality due to cannibalism in laying hens is a worldwide economic, health, and welfare problem, occurring in all types of commercial poultry housing systems [1]. Due to the likely prohibition of beak-trimming in the European Union in the near future, this problem will increase if no further actions are taken, and, therefore, needs to be solved urgently.

One of the possibilities is to use genetic selection [2,3]. However, selection for lower mortality has not been very effective in most cases [4]. First, heritabilities of mortality are low, ranging between 3.2% and 9.9%, leading to low accuracy [5-9]. Second, censoring is high (animals still alive at the end of the testing period have no record on survival time) [9], leading to low accuracy as well. Third, traditional methods for selection against mortality can lead to unfavourable response to selection, because these methods ignore the social effect an individual has on it's group members (so-called social interactions) [2,10-12].

Heritabilities for survival traits are often estimated using a linear animal model [8,13]. However, a linear animal model does not take into account the fact that some animals are still alive at the end of the testing period (so-called censored records), for these animals the true survival days is unknown. Furthermore, linear models do not properly account for the nature of survival data, because survival data are usually heavily skewed [14]. Survival analysis [15] appropriately accounts for both censoring and non-normality in the data. Survival analysis is used to examine either the length of time an individual survives or the length of time until an event occurs. Models for survival analysis can be built from a hazard function, which measures the risk of an event to occur, given that the individual has survived up to time *t *[14,16].

Social interactions occur when individuals are kept together in a group. Wolf [17] has mentioned that the environment provided by group members is often the most important component of the environment experienced by an individual in that group. There is clear evidence that social interactions contribute to the heritable variation in traits [2,8,13,17-21]. For instance, social interactions have a substantial genetic effect on mortality due to cannibalism [8,13,17,20,22-26]. Bijma et al. [13] and Ellen et al. [8] have found that ^1^/_3 _to ^2^/_3 _of the heritable variation in survival days is due to social interactions. To reduce mortality due to cannibalism, the classical model for a given genotype must be extended to consider not only the individuals' direct effect of its own genes, but also the associative genetic effect of the individual on the phenotypes of its group members [10]. Muir [2] has clearly shown that selection methods targeting both direct and associative genetic effects (group selection) results in a decrease in mortality due to cannibalism in laying hens, whereas selection based on only the direct genetic effect (individual selection) results in an increase in mortality [27]. Furthermore, Muir [20] has found that, in Japanese quail, group selection results in decreased mortality and increased bodyweight. However, so far associative genetic effects have not been implemented in existing software for survival analysis. To analyse data on mortality due to cannibalism, a solution might be to combine survival analysis and a linear animal model including associative effects.

Ducrocq et al. [28] have proposed a two-step approach for multiple trait evaluation of longevity and production traits in dairy cattle, which faces similar problems. The two-step approach is a combination of survival analysis and a linear animal model. In the first step, survival analysis is performed to compute the so-called pseudo-records and their associated weights. Pseudo-records can be regarded as the result in the data of a linearization of the model. When analysed with a simple linear animal model, pseudo-records weighted appropriately lead to the same estimated genetic values as the initial survival model used to compute them. In the second step, genetic parameters on pseudo-records with their associated weights are estimated using a linear animal model.

In this paper, we apply a similar two-step approach to estimate genetic parameters for direct and associative effects on survival time in laying hens. In the second step, we will use the linear animal model including associative effects to estimate genetic parameters [8,13,20]. For the remaining part of the paper, we will refer to the linear animal model including associative effects as LAM and to the two-step approach as 2STEP. Cross validation will be used to compare 2STEP with LAM [8,13]. LAM was applied directly to estimate genetic parameters for social effects on observed survival days. For the cross validation, the predicted hazard rate will be estimated using 2STEP and the predicted phenotype will be estimated using LAM. To judge the performance of both methods, predicted phenotypes or hazard rates will be compared with the observed phenotype.

## Methods

For this study, the same data were used as described in Ellen et al. [8]. The main characteristics are summarized below and further details are in [8].

### Population and housing

Data of three purebred White Leghorn layer lines from Institut de Sélection Animale B.V., a Hendrix Genetics company, were used in this study. The three lines were coded: W1, WB, and WF. For each line, observations on survival time of a single generation were used. Chickens of each line were hatched in two batches, each batch consisting of four age groups, differing by two weeks each. All chickens had intact beaks.

When the hens were on average 17 weeks old, they were transported to two laying houses with traditional four-bird-battery cages. Each batch was placed in another laying house. In both laying houses, the 17-week-old hens were allocated to laying cages, with four birds of the same line and age in a cage. The individuals making up a cage were combined at random. In both laying houses, cages were grouped into eight double rows. Each row consisted of three levels (top, close to the light; middle; and bottom). A feeding trough was in front of the cages, and each pair of back-to-back cages shared two drinking nipples.

### Pedigree

Sires used for both laying houses were largely the same while dams were different. For all three lines, sires and dams were mated at random. Each sire was mated to approximately eight dams, and each dam contributed on average 12.3 female offspring. Five generations of pedigree were included in the calculation of the relationship matrix (**A**). To avoid pedigree errors, hens with unknown identification or double identification were coded as having an unknown pedigree (*n *= 101). The observations on these hens were included in the analysis to better estimate fixed effects.

### Data

All hens were observed daily. Dead hens were removed from the cages and not replaced, and wing band number and cage number were recorded. The study was ended when hens were on average 75 weeks old. For each hen, information was collected on survival and number of survival days. Survival was defined as alive or dead (0/1) at the end of the study. From these data, the survival rate was calculated as the percentage of laying hens still alive at the end of the study. Survival days were defined as the number of days from the start of the study (day of transport to laying houses) till either death or the end of the study. Hens that died before the end of the study were referred to as a failure (event = 1), whereas hens still alive at the end of the study were referred to as censored (event = 0). In total, 196 hens were removed from the study, due to reasons other than mortality. These hens were referred to as censored (event = 0). For the statistical analysis, 6,276 records were used for line W1; 6,916 for line WB; and 3,588 for line WF.

### Data analysis

Data were analysed separately for each line. Two methods to estimate genetic parameters were compared: 1) LAM, a linear animal model including direct and associative effects applied directly to the observed survival days; this procedure is described in detail in [8], and 2) 2STEP, a two-step approach [29]. In the first step of 2STEP, data were analysed using survival analysis as implemented in the survival kit V5 [30], to produce pseudo-records as defined below. Survival analysis allows the combination of information from hens still alive at the end of the study (censored records) as well as hens that died (uncensored records). In the second step, genetic parameters for direct and associative effects on pseudo-records were estimated using a linear animal model [8,13], implemented in ASReml [31].

#### *Step 1: Survival analysis*

Data were analysed using the Cox animal model [32]. The Cox model can deal with non-linearity, censoring, and non-normal residuals. The model included a fixed effect for each combination of laying house, row, and level, and for average survival days in the back cage to account for a possible effect of the back neighbours [8]. Age was fully confounded with laying house and row and, therefore, not included as a fixed effect. All the fixed effects were significant.

Using survival analysis results in a breeding value (*a_i _*) and an associated weight (*ω_i_*) for each hen *i*. It can be shown that *ω_i _*is the estimated cumulative risk of animal *i *from time 0 to censoring time or death, and is therefore a function of the (possibly censored) length of life of hen *i*, her censoring code (*δ_i _*= 0/1), and the fixed effects in the model [29]. The pseudo-record for survival time of animal *i *was [33]:(1)

where *δ_i _*is the censoring code of individual *i *(*δ_i_*= 1 if animal *i *is uncensored; *δ_i _*= 0 if animal *i *is censored); *a_i _*is the estimated direct breeding value of individual *i*; and *ω_i_* is the associated weight of individual *i*. Pseudo-records are functions of the data and of the effects estimated in the survival model, such that when a straightforward BLUP animal genetic evaluation is applied on these pseudo-records, the same estimated breeding values are obtained as in the initial survival model.

To verify 2STEP, pseudo-records with appropriate weights were analysed to estimate breeding values with a univariate BLUP animal model, with a heterogeneous residual variance  for animal *i*. The correlation between the estimated breeding values of 2STEP and the estimated breeding values of the survival analysis was calculated [29]. As expected, this correlation was one and the estimated breeding values were the same. Thus the computation of pseudo-records in 2STEP was correct.

#### *Step 2: Associative effects model*

To estimate variances and covariances for direct and associative effects, using the pseudo-records and associated weights from step 1, the model of Muir [20] and Bijma et al. [13] was used:(2)

where **y **is a vector of the pseudo-records ; **a**_D _is a vector of direct breeding values, with incidence matrix **Z**_D_ linking observations on individuals to their direct breeding value; **a**_S _is a vector of associative breeding values, with incidence matrix **Z**_S _linking observations on individuals to the associative breeding values of their group members (i.e., individuals in the same cage); and **e **is a vector of residuals, where . A weighted analysis was performed using the associated weight (*ω_i_*) and the !WT statement in ASReml [31] and fixing  to one [28].

The covariance structure of genetic terms is ,

where , in which  is the direct genetic variance,  is the associative genetic variance, and  is the direct-associative genetic covariance. Bijma et al. [13] have shown that residuals of group members are correlated due to non-genetic associative effects. The covariance structure of the residual term, **e**, is given by , where *R_ij _*= 1 when *i *= *j*, *R_ij _*= *ρ *when *i *and *j *are in the same group (*i *≠ *j*), and *R_ij _*is zero otherwise. The value of *ρ *was estimated in the analysis, using a CORU statement in the residual variance structure in ASReml [31].

#### *Heritable variation*

When social interactions exist among individuals, each individual interacts with *n *- 1 group members. In this study, *n *= 4. The total heritable impact of an individual on the population, referred to as its total breeding value (TBV), equals the sum of its direct breeding value and *n *- 1 times its associative breeding value: TBV*_i _*= A_D_, *_i _*+ (*n *- 1) A_S,*i *_[20]. The total heritable variation equals the variance of the TBV among individuals, [13,34]. With unrelated group members, the phenotypic variance equals . The total heritable variance expressed relative to the phenotypic variance equals . The *T*^2^ expresses the total heritable variance relative to the phenotypic variance and is, therefore, a generalisation of the conventional *h*^2 ^to account for social interactions.

#### *Cross validation*

We compared 2STEP to LAM using cross validation [35]. With cross validation, known phenotypes are set to missing and their value is predicted and compared with their observed phenotype. Validation was applied separately to each of the three lines. For this purpose, a random number was allocated to each cage within a fixed effect class. For each line, phenotypes of animals from 20% of the cages from each fixed effect class were set to missing, which resulted in five subsets, each containing 80% of the data. In this way, each cage was once removed from the total dataset, and each fixed effect class was present in all five subsets. The phenotypes set to missing were predicted using a combination of the direct breeding value of the individual itself and the associative breeding values of its group members  of either 2STEP or LAM.

Comparing the predicted phenotypes of both methods is difficult for two reasons. First, a scale difference exists between estimated breeding values (EBV) of 2STEP and EBV of LAM. EBV of LAM are on the observed scale for survival days, whereas EBV of 2STEP are on the hazard rate scale. Transforming EBV of 2STEP into survival days is somewhat difficult, because the transformation is non-linear. Therefore, the predicted phenotypes using 2STEP are on the hazard rate scale, whereas the predicted phenotypes using LAM are on the observed scale for survival days. Second, in our dataset approximately 50-70% of the data were censored (animals that were still alive at the end of the testing period). These animals do not have an observed phenotype. In other words, a large proportion of the "observed" phenotypes is censored, and cannot be compared directly to their prediction. However, we know that their observed phenotypes are larger than those of animals that are not censored, which is highly relevant information.

To deal with these two difficulties, we used two approaches to evaluate both methods. The first approach is based on using groups of animals rather than single individuals. In this approach, for each subset and method, 25% of the animals with the best predicted phenotypes or hazard rates were selected as the best groups (best refers to animals with the highest predicted phenotypes using LAM or lowest predicted hazard rates using 2STEP), and 25% of the animals with the worst predicted phenotypes or hazard rates were selected as the worst groups. The Kaplan-Meier estimate of the survival curve was plotted for the best and worst groups based on the observed phenotypes. It was expected that the best groups would yield the best Kaplan-Meier estimate of the survival curve, whereas the worst groups would yield the worst one. Moreover, for both methods the mean observed survival days were calculated for the best and worst groups. From these, the difference in survival days between the best and worst group was calculated. For the best groups the percentage of overlapping animals, between 2STEP and LAM, was calculated.

To quantify the contribution of social effects to the predicted phenotype, phenotypes or hazard rates were predicted using different EBV: 1) classical BV (CBV); 2) direct BV of the individual itself (DBV = *A_D, i _*); associative BV of the group members () and a combination of the direct BV of the individual itself and the associative BV of its group members  CBV were estimated using a classical linear animal model given in [8] or using survival analysis (first step of 2STEP). DBV, SBV and DSBV were estimated using LAM or 2STEP.

The second approach is based on using ranks of individuals rather than their observed phenotypes. In this approach, the rank correlation between the observed phenotype and predicted phenotype or hazard rate and between predicted phenotype and predicted hazard rate was calculated. Due to the scale difference and censoring, Pearson correlations cannot be used. However, in both methods animals with the highest predicted phenotype (LAM) or lowest predicted hazard rate (2STEP) have the highest expected value for observed survival days. Therefore, the rank correlation between predicted and observed values can be used for both methods. Hence, the use of rank rather than Pearson correlation solves the scale issue. The remaining problem is animals with censored records, which have an unknown rank for the observed phenotype. However, the fact that animals were censored, represents an important information because those animals had the highest observed survival days. Animals with known phenotypes have a rank 1 through *n*, whereas censored animals have rank *n+*1 through *N*, but with an unknown order. For the censored animals, we assumed that their ranks are in random order between *n+*1 and *N*, so that the rank among the censored animals does not contribute to the estimated rank correlation. In this case, the rank correlation can be calculated by giving censored animals the average rank of all the censored animals [see Additional file 1]. In this way, we use the information that animals were censored, but make as little assumptions as possible about their order.

Before calculating rank correlations, observed phenotypes were corrected for the fixed effects [8], . Next, for the 20% missing data, the correlation was calculated between the rank of the observed phenotypes corrected for the fixed effects, accounting for censoring as described above, and the rank of the predicted phenotypes or hazard rate: . In this expression,  denotes the predicted phenotype in case of LAM and the predicted hazard rate times -1 in case of 2STEP. Note, the  of individual *i *is the sum of the estimated direct breeding value (or hazard rate) of hen  and the estimated associative breeding values (or hazard rates) of its group members . Furthermore, to quantify similarity of both methods, the rank correlation between the  of 2STEP and the  of LAM was calculated.

**Figure 1:**
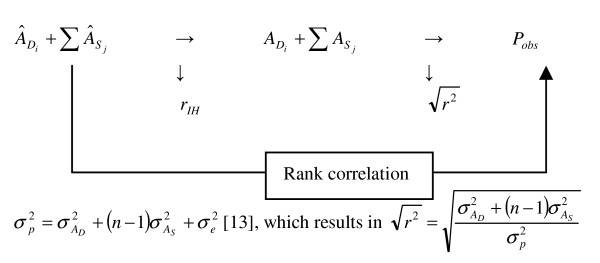
**Approximate accuracy**.

The rank correlation between predicted and observed phenotypes depends not only on the accuracy of the estimated breeding values underlying the predictions, but is also affected by non-genetic components of the observed phenotype. If breeding values underlying predicted phenotypes were estimated with full accuracy, the correlation between predicted and observed phenotypes would be equal to the square root of the proportion of phenotypic variance explained by breeding values, . For any accuracy of predicted breeding values (*r*_*IH*_), the expected correlation between predicted and observed phenotypes would be equal to  (Figure 1), where *r_IH _*is the accuracy of . Because animal breeders are interested in predicting breeding values rather than phenotypes, we calculated an approximate accuracy as . Hence,  represents the approximate accuracy with which the genetic components underlying the observed phenotype, , were predicted. This accuracy is only approximate because it refers to the ranks rather than the phenotypes, and because the prediction from 2STEP refers to the scale of the hazard rate rather than the observed phenotype. For line W1 = 0.32, for line WB  = 0.37, and for line WF  = 0.17, when using the genetic parameters (see Table 1) given in Ellen et al. [8].

**Table 1:** Estimates of genetic parameters for direct and associative effects on survival time in three layer lines using 2STEP or LAM [8]

	W1		WB		WF	
	2STEP	LAM	2STEP	LAM	2STEP	LAM
	0.31 ± 0.05	915	0.30 ± 0.05	1,917	0.12 ± 0.06	246
	0.041 ± 0.01	134	0.028 ± 0.01	273	0.049 ± 0.02	60
	0.77 ± 0.13	2,490	0.44 ± 0.09	3,007	0.81 ± 0.26	910
	1.44 ± 0.06	12,847	1.38 ± 0.05	20,111	1.27 ± 0.08	13,999
*T*^2^	0.53 ± 0.08	0.19	0.32 ± 0.06	0.15	0.64 ± 0.17	0.06
*r_A_*	0.13 ± 0.15	0.18	-0.20 ± 0.14	-0.31	0.55 ± 0.28	0.11
*ρ*	-0.003 ± 0.0003	0.08	-0.005 ± 0.0001	0.08	-0.004 ± 0.0003	0.10

and  are estimates of direct genetic variance and associative genetic variance;  is the total heritable variance: ;  is the phenotypic variance: , where  = 1; *T*^2 ^expresses the total heritable variance relative to the phenotypic variance: ; *r_A _*is the genetic correlation between direct breeding value and associative breeding value; *ρ *is the correlation between the residuals of group members

## Results

### Survival

**Figure 2:**
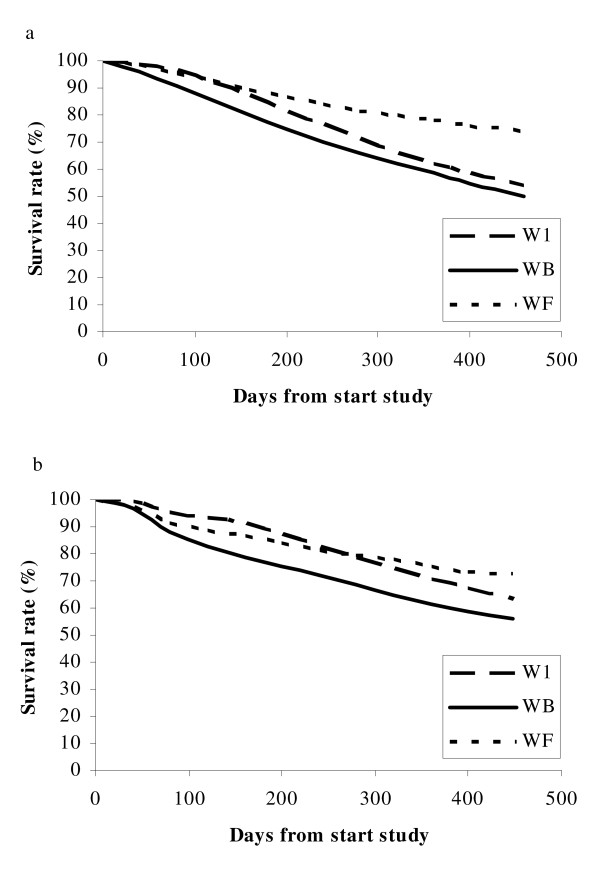
**Survival curve of the three layer lines**. Survival curve is shown for the three lines W1, WB, and WF housed in laying house 1 (a) and laying house 2 (b).

The Kaplan-Meier estimate of the survival function [36] was plotted for the survival of the three layer lines (Figure 2). The survival function represents the proportion of laying hens that survived up to time *t*. The survival rate differed significantly between lines in both laying houses (p < 0.01). Line WF showed the highest survival rate i.e. 74.6%, whereas line WB showed the lowest survival rate i.e. 52.9%.

### Genetic parameters

The estimated genetic parameters for direct and associative effects using 2STEP are given in Table 1. For all three lines, both the direct genetic variance  and the associative genetic variance  were significantly different from zero. The was lowest in line WF and highest in line W1, ranging from 0.12 through 0.31, whereas the  was lowest in line WB and highest in line WF, ranging from 0.028 through 0.049. The total heritable variance  ranged from 0.44 (WB) through 0.81 (WF) and was significantly different from zero. Line WB showed the lowest total heritable variance in survival days expressed relative to the phenotypic variance (*T*^2 ^), whereas line WF showed the highest *T*^2 ^, ranging from 32% through 64%. The estimated genetic correlation between direct breeding value and associative breeding value (*r_A _*) was positive but not significantly different from zero in line W1 (0.13) and *A *line WF (0.55), and negative and not significantly different from zero in line WB (- 0.20). Table 1 shows also the genetic parameters using LAM [8].

### Cross validation

**Figure 3:**
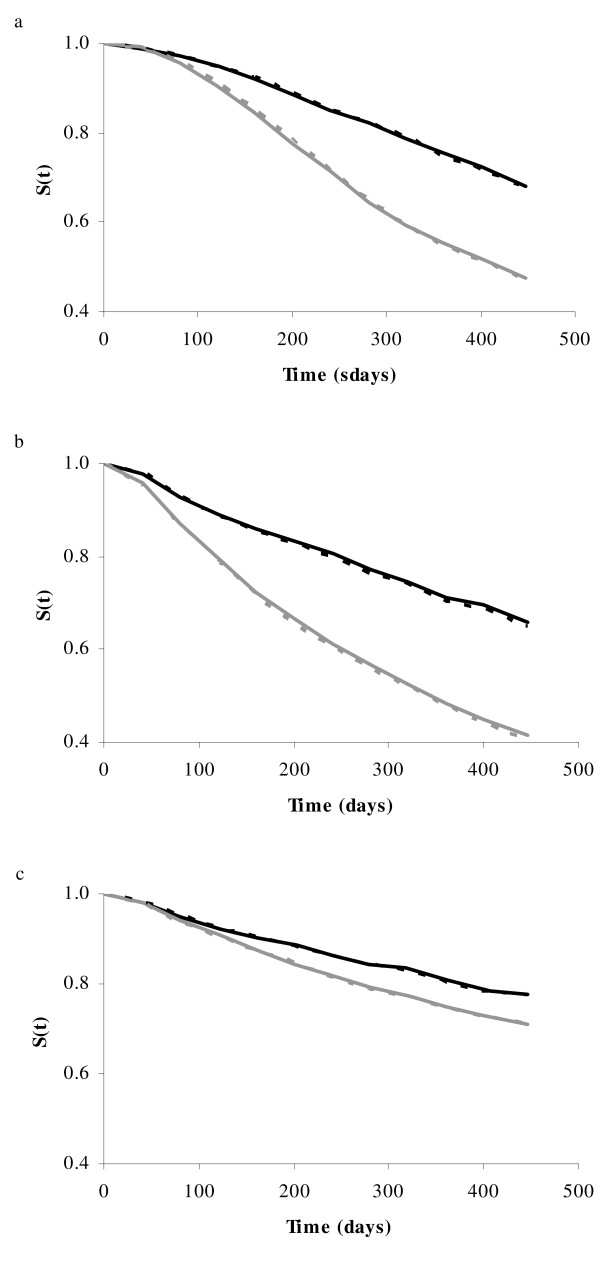
**Survival curves using 2STEP or LAM**. Kaplan-Meier non-parametric estimate of the observed survival curve of two extreme groups, based on the predicted phenotypes (LAM) or predicted hazard rates (2STEP). For each subset and method, phenotypes or hazard rates were predicted based on DSBV. 25% of the animals with best predicted phenotypes or hazard rates were selected as the best groups (best refers to animals with the highest predicted phenotypes using LAM or lowest predicted hazard rates using 2STEP), and 25% of the animals with the worst predicted phenotypes or hazard rates were selected as the worst groups. Black solid line: best group using 2STEP; black dotted line: best group using LAM; gray solid line: worst group using 2STEP; gray dotted line: worst group using LAM. Results are averages of five subsets, each containing 20% of the data. Figures represent line W1 (a), WB (b), and WF (c).

Figure 3 shows the Kaplan-Meier estimate of the survival curves for the groups with the best and worst predicted phenotypes, using either 2STEP or LAM. As expected, for all three layer lines, groups with the best predicted phenotypes or hazard rates yielded the best observed survival curves, whereas groups with the worst predicted phenotypes or hazard rates yielded the worst observed survival curves. Both line W1 and WB showed a large difference in survival curves between the best and worst groups, whereas this difference was smaller in line WF. For all three lines, there was hardly any difference in survival curves between 2STEP and LAM. Meaning that both predicted phenotypes or hazard rates are good indicators for observed survival days. Table 2 shows the average survival days of the best and worst group for both methods and each line. Again, there was hardly any difference in average survival days between 2STEP and LAM. Furthermore, Table 2 shows the difference in survival days between the best and worst group. The difference was largest in line WB (67 days, for both methods) and smallest in line WF (16 days, for both methods). These results are in accordance with the difference in survival curves (Figure 3).

**Table 2:** Mean survival days of best and worst groups using 2STEP or LAM for three layer lines

	W1		WB		WF	
	2STEP	LAM	2STEP	LAM	2STEP	LAM
Mean	354 ± 2		326 ± 2		375 ± 2	
Best	377 ± 3	377 ± 3	359 ± 2	357 ± 3	384 ± 3	383 ± 5
Worst	327 ± 2	327 ± 3	292 ± 6	290 ± 6	368 ± 7	367 ± 5
Difference	50	50	67	67	16	16

Best group = 25% of the animals with best predicted phenotypes (LAM) or hazard rates (2STEP); worst group = 25% of the animals with worst predicted phenotypes or hazard rates; difference = mean survival days of best group - mean survival days of worst group; results are averages of five subsets, each containing 20% of the data

**Figure 4:**
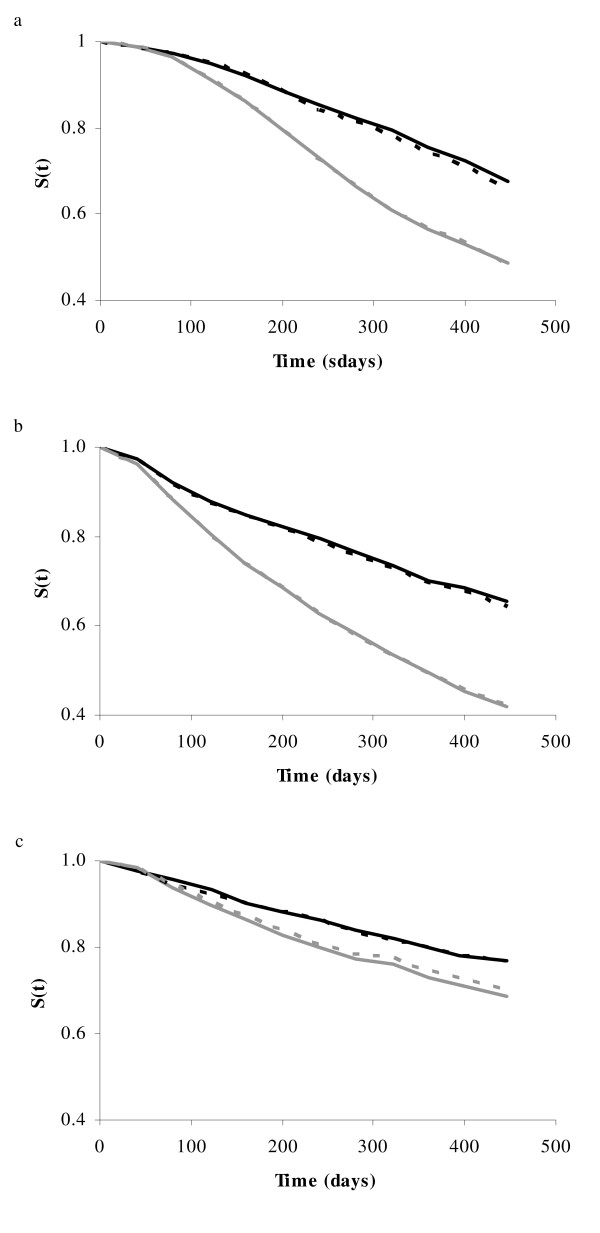
**Survival curves based on CBV, using survival analysis or classical linear animal model**. Kaplan-Meier non-parametric estimate of the observed survival curve of two extreme groups, based on the predicted phenotypes (classical linear animal model) or predicted hazard rates (survival analysis). For each subset and method, phenotypes or hazard rates were predicted based on CBV. 25% of the animals with best predicted phenotypes or hazard rates were selected as the best groups (best refers to animals with the highest predicted phenotypes using classical linear animal model or lowest predicted hazard rates using survival analysis), and 25% of the animals with the worst predicted phenotypes or hazard rates were selected as the worst groups. Black solid line: best group using survival analysis; black dotted line: best group using classical linear animal model; gray solid line: worst group using survival analysis; gray dotted line: worst group using classical linear animal model. Results are averages of five subsets, each containing 20% of the data. Figures represent line W1 (a), WB (b), and WF (c).

To quantify the contribution of social effects to the predicted phenotype or hazard rate, the phenotype or hazard rate is predicted using different breeding values, CBV, DBV, SBV and DSBV. Again, for each of the three lines, 25% of the animals with best predicted phenotypes or hazard rates were selected as the best group, and 25% of the animals with the worst predicted phenotypes or hazard rates were selected as the worst group. Table 3 shows the difference in survival days between the best and worst groups for both methods and each line. Besides, the Kaplan-Meier estimate of the survival curves for the groups with the best and worst predicted phenotypes based on CBV, using the two methods, is given in Figure 4. For both lines WB and W1, animals selected on predicted phenotypes or hazard rates using DSBV gave the largest difference in survival days between the best and worst group. Furthermore, for both lines, using CBV to predict the phenotype or hazard rate gave similar difference in survival days as the DBV (approximately 45 days for line W1 and 58 days for line WB). For line WF, the difference in survival days depends on the method used. For 2STEP, the difference was largest when CBV was used, whereas for LAM the difference was largest when DSBV was used. Furthermore, for each layer line, the overlap of animals in the best group between 2STEP and LAM was calculated. The average overlap was 85% for both lines W1 and WB, and 74% for line WF. These results show that a large proportion of the animals selected for the best group, using either 2STEP or LAM, are the same.

**Table 3:** Difference in survival days between best and worst groups using 2STEP or LAM for three layer lines

	W1		WB		WF	
	2STEP	LAM	2STEP	LAM	2STEP	LAM
CBV	46	43	58	57	19	14
DBV	45	43	59	57	15	11
SBV	25	26	32	33	12	9
DSBV	50	50	67	67	16	16

Best group = 25% of the animals with best predicted phenotypes or hazard rates; worst group = 25% of the animals with worst predicted phenotypes or hazard rates; phenotypes or hazard rates are predicted using CBV, DBV (*A_D, i_*), , or

The rank correlations between the observed phenotype, adjusted for fixed effects and censoring, and the predicted phenotype, , are given in Table 4. The rank correlations were low and approximately the same for both methods. For line W1 (0.149 vs. 0.144) and WB (0.174 vs. 0.170), they were slightly, but not significantly, better for 2STEP, whereas for line WF (0.039 vs. 0.042) it was slightly, but not significantly, better for LAM. The rank correlations between the predicted phenotype using 2STEP and LAM were high and ranged from 0.879 (line WF) through 0.962 (line WB). These results are in line with the survival curves (Figure 3). Furthermore, approximate accuracies were calculated (Table 4) and were moderate, and approximately the same for both methods.

**Table 4:** Rank correlation and approximate accuracy based on 2STEP or LAM for three layer lines

	Rank correlation			Approximate accuracy		
	W1	WB	WF	W1	WB	WF
2STEP	0.149 ± 0.011	0.174 ± 0.020	0.039 ± 0.017	0.47	0.47	0.22
LAM	0.144 ± 0.010	0.170 ± 0.020	0.042 ± 0.012	0.45	0.46	0.24
2STEP; LAM	0.954 ± 0.003	0.962 ± 0.004	0.879 ± 0.007	-	-	-

Rank correlation is calculated between observed phenotypes and predicted hazard rates of 2STEP, between observed phenotypes and predicted phenotypes of LAM, and between predicted phenotypes and predicted hazard rates (2STEP; LAM); observed phenotype = phenotype corrected for fixed effects ; predicted phenotype or hazard rate () = sum of estimated direct breeding value of hen  and estimated associative breeding values of its group members ; approximate accuracy = *r*_*IH *_= , where *corr *is the rank correlation between observed phenotypes and predicted phenotypes or hazard rates, and  = 0.32 for line W1, 0.37 for line WB, and 0.17 for line WF, when using the genetic parameters given in Ellen et al. [8]; results are averages of five subsets, each containing 20% of the data

## Discussion

We have estimated genetic parameters for direct and associative effects using 2STEP, combining survival analysis and a linear animal model including associative effects. Using 2STEP, the total heritable variance, including both direct and associative genetic effects, expressed as the proportion of phenotypic variance (*T*^2 ^), ranged from 32% (line WB) through 64% (line WF). Using 2STEP, *T*^2 ^is substantially larger than using LAM. However, results of the cross validation do not show any difference between the two methods. Using cross validation, we showed that the difference in survival days between two extreme groups is largest when selecting on DSBV. Furthermore, we showed that social genetic effects contribute substantially to the difference in survival days (Table 3). These results indicate that there could be quite some gain in survival days when selecting on the combination of the direct breeding value and the associative breeding values of the group members (DSBV). Comparing genetic parameters of 2STEP and LAM is not straightforward. For 2STEP, genetic parameters are given on the hazard rate scale, the probability that an animal has a failure at a given time *t*, whereas genetic parameters of LAM are on the observed scale for survival days. The difference in genetic parameters between 2STEP and LAM originates from the fact that there is a scale difference, just like the difference in heritabilities for a 0/1-trait between linear and threshold models [37]. Using 2STEP, the total heritable variance is 1.5 to 7-fold greater than the classical direct genetic variance using survival analysis. For both lines W1 and WB, this increase in total heritable variance is comparable with results found using LAM [8,13] (1.5 to 3-fold). For line WF, the increase is much larger using 2STEP (7-fold) than using LAM (3-fold), which could be due to the fact that censoring is higher in line WF and 2STEP takes this better into account.

Theoretically, 2STEP would be a better method to analyse survival data, based on fewer assumptions known to be incorrect. We used two approaches to compare the two methods; selection of animals with the best and worst predicted phenotypes or hazard rates and the rank correlation between the predicted phenotypes or hazard rates and observed phenotypes. Both approaches show that there is hardly any difference between 2STEP and LAM. This applies to all three lines. At first glance, the difference in *T*^2 ^between both methods might suggest that using 2STEP would yield greater genetic improvement than using LAM [38]. However, as explained above, this difference arises from a difference in scale. The cross validation clearly demonstrates that both methods yield very similar rates of genetic improvement.

Note that the rank correlation is low for all three lines, whereas the approximate accuracy is moderate for lines W1 and WB and low to moderate for line WF. Even though the approximate accuracy seems low, it is in accordance with the accuracy for methods that contain only half- or full-sib information (at least for lines W1 and WB) [11,39]. Furthermore, a high rank correlation was found between the predicted hazard rates of 2STEP and the predicted phenotypes of LAM. Using selection of the best and worst predicted phenotypes or hazard rates, approximately 80% of the animals selected for the best predicted phenotypes were overlapping between 2STEP and LAM. Based on the high overlap of animals between 2STEP and LAM and the similar rank correlation, it implies that, for both methods, a similar genetic progress will be achieved.

We made a number of assumptions in the cross validation, when using the rank correlation, that may have affected the results. First, observed phenotypes were corrected for fixed effects using LAM, which may have favoured LAM compared to 2STEP. Second, when calculating the rank correlation, we assumed that ranks of censored records were in random order. This will probably not be true if censored animals were given the opportunity to actually produce a record. Alternatively, we could have used the ranks of the uncensored records only. However, in that case we would have ignored the information that the censored records are actually the "best records".

For all three layer lines, censoring occurred at the same time, at the end of the study. It could be that when censoring occurs at different times during the study period, differences may occur between the two methods. To investigate this, 50% of the censored records of line W1 were censored half way the study period (at 200 days). Again cross validation was used to compare the two methods. For both methods, the difference in survival days between the group with best predicted phenotypes or hazard rates and the group with the worst predicted phenotypes or hazard rates was calculated. For 2STEP the difference was 25.9 days, whereas for LAM the difference was 15.7 days. This indicates that, when the censoring times differ between individuals, 2STEP can better identify the genetically superior individuals than LAM. Thus both methods are practically equivalent when all animals are censored at the same survival time; with variation in censoring time, the 2STEP is superior.

## Conclusion

This study shows that it is possible to use 2STEP, a combination of survival analysis and a linear animal model including associative effects, to estimate genetic parameters for the direct and associative effects on survival time in laying hens. We used cross validation to compare 2STEP with LAM. Based on the results in this paper, we can conclude that both 2STEP and LAM are practically equivalent when all animals are censored at the same survival time. Cross validation showed that selecting on a combination of the direct BV and the associative BV of the group members (DSBV) gave the largest difference in survival days between two extreme groups.

Furthermore, this study showed that social genetic effects contribute substantial to the difference in survival days between two extreme groups, which means that social genetic effects do exist.

## List of abbreviations

2STEP: two-step approach; LAM: linear animal model including direct and associative effects; CBV: classical breeding value; DBV: direct breeding value: SBV: associative breeding value; DSBV: combination of the direct breeding value of the individual itself and the associative breeding value of its group members.

## Competing interests

The authors declare that they have no competing interests.

## Authors' contributions

EDE performed the data analysis and the cross validation, wrote and prepared the manuscript for submission. VD helped with the data analysis and cross validation and reviewed the manuscript. BJD helped with the data analysis and reviewed the manuscript. RFV helped with the data analysis and reviewed the manuscript. PB was the principal supervisor of the study and assisted with data analysis, cross validation and preparation of the manuscript. All authors read and approved the manuscript.

## Supplementary Material

Additional file 1 **Example and mathematical proof of rank correlation with censoring**. Click here for file
