# Supplementary material for: Genetic parameters for social effects on survival in cannibalistic layers: Combining survival analysis and a linear animal model
Source: Genet Sel Evol. 2010 Jul 7;42(1):27. doi: 10.1186/1297-9686-42-27 (PMC2912826; doi:10.1186/1297-9686-42-27)
Supplement: Additional file 1 — Example and mathematical proof of rank correlation with censoring. [file 1297-9686-42-27-S1.PDF]

Additional files

Additional file 1 – Example and mathematical proof of rank correlation with censoring

Suppose we have a dataset of 200 individuals with 60% censoring. The phenotype is known of 80 individuals, whereas the phenotype is unknown of 120 individuals. The individuals with known phenotypes have rank 1 through 80, whereas the individuals with unknown phenotypes have rank 81 through 200, but in unknown order. For the censored phenotypes, we assumed that their rank is in random order between 81 and 200, so that the rank among the censored individuals does not contribute to the estimated rank correlation. In this case, the rank correlation can be calculated by giving censored individuals the average rank of 140.5 (in this example). In this way, we utilize the information that animals were censored, but make as little assumptions as possible about their order.

Let k denote the ranks of the observed phenotypes, k = (1, N), with $k \leq n$ representing the known records, and k = (n + 1, N) representing the unknown ranks of the censored records. Thus k is known for $k \leq n$, and k is unknown for k = (n + 1, N). Moreover, let $\hat{k}$ denote the ranks of the predicted values, with $\hat{k}$ = (1, N), and all $\hat{k}$ are known. The problem is to calculate the correlation between the ranks k and $\hat{k}$, taking into account censoring. Both k and $\hat{k}$ range between 1 and N, so we can use $Var(k) = Var(\hat{k})$. Thus the rank correlation equals $\rho = Cov(k,\hat{k})/Var(\hat{k})$. Next, $Cov(k,\hat{k})$ = $\frac{1}{N-1}\left[\sum_N k\hat{k} - \frac{1}{N}\sum_N k \sum_N \hat{k}\right]$. Since all ranks range from 1 through N, the second term in square brackets is known. The first term can be split into a known component, and a component including the censored records given by $\sum_{j=n+1}^{N} k_j \hat{k}_j$, where k_j is censored.

Taking the expectation, assuming that ranks of censored records are in random order

between n + 1 and N, gives $E\left[\sum_{j=n+1}^{N} k_j \hat{k}_j\right] = \sum_{j=n+1}^{N} E(k_j \hat{k}_j) = \sum_{j=n+1}^{N} \hat{k}_j E(k_j)$, meaning that we can use the mean rank of the censored records, $\bar{k}_{censored} = E(k_j \mid j = n+1, N) = (n+1+N)/2$. Therefore, we calculated the rank correlation as $\rho = Cov(k, \hat{k})/Var(\hat{k})$, substituting $k = \bar{k}_{censored}$ in the calculation of the censored elements of $\sum_N k\hat{k}$.
